# Rationale and Design of Heart Failure Prevalence and Evolution of Heart Failure in Diabetes Mellitus Type II Patients at High Risk (HF-LanDMark Study)

**DOI:** 10.3390/jcm12196319

**Published:** 2023-09-30

**Authors:** John Parissis, Christos Georgiou, Vasiliki Bistola, Apostolos Karavidas, Vassilios P. Vassilikos, John Kanakakis, Periklis Davlouros, Dimitrios N. Tziakas, Ioannis P. Alexanian, George Kochiadakis, Filippos Triposkiadis, Haralambos Karvounis, Dimitrios Gourlis, Nikolaos Papoutsidakis, Effie Polyzogopoulou, Charalambos Vlachopoulos

**Affiliations:** 1 Emergency Medicine Department, Attikon University Hospital, National and Kapodistrian University of Athens, Rimini 1, Chaidari, 12461 Athens, Greece; christosgeorgiou_md@yahoo.gr (C.G.); effiep@live.com (E.P.); 2 Heart Failure Unit, 2nd Department of Cardiology, Attikon University Hospital, National and Kapodistrian University of Athens, Rimini 1, Chaidari, 12461 Athens, Greece; 3 Department of Cardiology, “G. Gennimatas” General Hospital, 11527 Athens, Greece; akaravid@yahoo.com; 4 3rd Cardiology Department, Hippokration General Hospital of Thessaloniki, 54642 Thessaloniki, Greece; vvassil@med.auth.gr; 5 Department of Clinical Therapeutics, University of Athens Medical School, 11527 Athens, Greece; jkanakakis@yahoo.gr; 6 Department of Cardiology, School of Medicine, University of Patras, 26504 Patras, Greece; pdav@med.upatras.gr; 7 Cardiology Department, Medical School, Democritus University of Thrace, 68100 Alexandroupolis, Greece; dtziakas@med.duth.gr; 8 Second Department of Cardiology, Evangelismos General Hospital of Athens, 10676 Athens, Greece; g_alexanian@hotmail.com; 9 Department of Cardiology, Heraklion University Hospital, 71500 Iraklio, Greece; kochiadg@gmail.com; 10 Department of Cardiology, Larissa University General Hospital, 41334 Larissa, Greece; ftriposkiadis@gmail.com; 11 1st Department of Cardiology, AHEPA Hospital, Aristotle University of Thessaloniki, 54636 Thessaloniki, Greece; hkarvounis@hotmail.com; 12 AstraZeneca Greece, Agisilaou 6-8, 15123 Maroussi, Greece; dimitris.gourlis@astrazeneca.com (D.G.); nikolaos.papoutsidakis@astrazeneca.com (N.P.); 13 First Cardiology Department, Hippokration General Hospital, School of Medicine, National and Kapodistrian University of Athens, 12461 Athens, Greece; cvlachop@otenet.gr

**Keywords:** type 2 diabetes mellitus, heart failure incidence, heart failure prevalence, quality of life, sodium–glucose cotransporter-2 inhibitors

## Abstract

(1) Background: Patients with diabetes mellitus (DM) are at increased risk for heart failure (HF). Accurate data regarding the prevalence of HF stages among diabetics in Greece are scarce. (2) Aim: The present study will examine the prevalence and evolution of HF stages among patients with type II DM (T2DM) diagnosed in the past 10 years, with no previous history of HF and at high CV risk, in Greece, as well as will explore the potential determinants of the development of symptomatic HF in these patients. (3) Methods: Through a non-interventional, epidemiological, single-country, multi-center, prospective cohort study design, a sample of 300 consecutive patients will be enrolled in 11 cardiology departments that are HF centers of excellence. Patients will be either self-referred or referred by primary or secondary care physicians and will be followed for up to 24 months. Demographic, clinical, echocardiography, electrocardiography, cardiac biomarkers (troponin, NT-proBNP) and health-related quality of life questionnaire data will be recorded as well as clinical events, including mortality, HF hospitalizations and HF-related healthcare resource utilization. The primary outcomes are the proportion of patients diagnosed with symptomatic HF (ACC/AHA Stage C) at enrolment in the overall study population and the proportions of patients with HF stages A, B and C, as well as by NYHA functional classification in the overall study population. (4) Conclusions: The HF-LanDMark study is the first epidemiological study that will assess the prevalence of HF among T2DM patients in Greece that could potentially enhance prompt therapeutic interventions shown to delay the development of HF in the T2DM patient population (HF-LanDMark, Clinical Trials.gov number, NCT04482283).

## 1. Introduction

Diabetes mellitus (DM) is a major public health problem, with over 400 million adults diagnosed worldwide. The prevalence of the disease is increasing, and, by 2040, the International Diabetes Federation (IDF) estimates that approximately 700 million adults will be diagnosed with DM [1]. Clinical and epidemiological data indicate that patients with DM are at high risk for adverse outcomes, such as myocardial infarction and other atherosclerosis-related cardiovascular (CV) events [2], as well as renal failure. Heart failure (HF) is a major contributor to cardiovascular (CV) morbidity and mortality in DM, with prevalence in type II DM (T2DM) up to 28% [3]. Risk factors for HF in DM include established CV disease, the presence of CV risk factors and chronic kidney disease [4,5]. Importantly, patients with both HF and DM have a higher risk of CV death or HF hospitalization than patients with either one of the diseases alone [6].

As per the ACC/AHA HF classification, patients with DM have, at least, stage A HF, i.e., are at risk for developing clinical HF. The distribution of DM patients in HF stages A/B/C was 48%, 36% and 16% in a study of patients of age ≥ 60 years with previously unrecognized HF [7]. Over a quarter of T2DM patients aged ≥60 years may have unrecognized clinical HF [8]. The risk of progression to symptomatic HF (stage C) from asymptomatic left ventricular diastolic dysfunction or systolic dysfunction (Stage B) is 8- and 2-fold higher than the respective risk of stage A [9]. The global longitudinal strain may independently predict incident HF among stage B patients [10,11]. Cardiac biomarkers, including high-sensitivity cardiac troponin T (hs-cTnT) and amino-terminal pro-B-type natriuretic peptide (NT-proBNP), can enhance the recognition of HF among at-risk individuals, including T2DM, when combined with traditional risk scores [12]. Recently, a biomarker risk score that included high-sensitivity troponin T, NT-proBNP, high-sensitivity C-reactive protein and left ventricular hypertrophy on electrocardiogram was shown to stratify HF risk in patients with dysglycemia (diabetes and pre-diabetes) and inform the allocation of HF prevention therapies in these patients [13].

The prevalence of DM among Greek adults based on data from the electronic prescription database has been previously reported as 8.2% [14]. However, accurate epidemiological data regarding the prevalence of HF stages among diabetics in Greece are scarce. The HF prevalence and evolution of HF in DM II patients at high risk (HF-LanDMark) epidemiological study aims to generate novel real-world data on the prevalence and evolution of HF stages in Greece and to explore potential determinants of the development of symptomatic HF, among patients with T2DM diagnosed in the past 10 years who are at high CV risk. Furthermore, it will attempt to measure the burden of HF- and CV-related healthcare resource utilization (HCRU) based on the HF stage and to capture the impact of symptomatic HF on patient-perceived health status.

## 2. Methods

### 2.1. Study Design

HF-LanDMark is an observational, non-interventional, epidemiological, single-country, multicenter, prospective cohort study that will examine the prevalence of previously undiagnosed symptomatic HF and the distribution of HF stages A/B/C in T2DM patients diagnosed within 10 years from enrolment, with no previous history of HF, who have high or very high CV risk (as outlined in 2019 European Guidelines on diabetes, pre-diabetes and CV diseases) [15].

This study received approval from the ethics committee of all participating departments (Ethics approval number 371/7-7-2020), and all patients will sign informed consent before enrolment.

### 2.2. Patient Population

Approximately 300 consecutive eligible patients, managed in real-life clinical settings in Greece, will be recruited in 11 Cardiology Departments that are HF centers of excellence. Patients will be either self-referred or referred by primary or secondary care physicians (endocrinologists, diabetologists, internists, other) to Cardiology Departments. Patients aged between 40 and 80 years with T2DM diagnosed within 10 years prior to enrolment who are at high or very high CV risk are eligible to be included in the study. High or very high CV risk is defined according to 2019 guidelines on diabetes, pre-diabetes and CV diseases from the European Society of Cardiology. Briefly, very high CV risk is defined as having either established CVD (ischemic heart disease (IHD), cerebrovascular disease (CeDV), peripheral arterial disease (PAD)) or other target organ damage (proteinuria, left ventricular hypertrophy (LVH) or retinopathy), or at least three major risk factors (age, hypertension, dyslipidemia, smoking and obesity (body mass index, BMI ≥ 30 kg/m^2^). High CV risk is defined as age ≥ 50 years and presence of at least one additional major risk factor. Recruitment will last up to 27 months. Each participating patient will be followed for up to 24 months or until death, withdrawal of consent or physician’s decision for patient withdrawal, whichever occurs earlier. Main exclusion criteria are diagnosis of T1DM, prior history of HF or treatment with SGLT2 inhibitor at the time of study enrolment because of the effect of this drug class to reduce new-onset HF in patients with T2DM. Detailed inclusion and exclusion criteria are listed in the Supplementary Materials.

### 2.3. Study Methods

**Figure 1:**
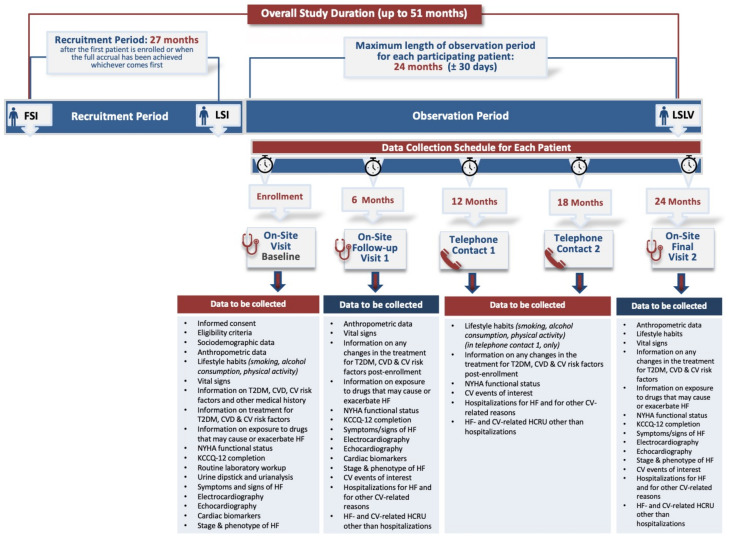
Study design and flow chart.

The study design and flow chart are presented in Figure 1. Patient follow-up visit frequency will be determined by the participating hospital-based investigators. However, study-related data will be collected at enrolment and at 6-, 12-, 18- and 24-month timepoints post-enrolment. Data collection at enrolment, and at 6 and 24 months post-enrolment will be performed in the context of on-site routine clinical visits at the hospital sites, whereas data collection at 12 and 18 months will be performed through telephone contacts.

### 2.4. Study Variables

Data collection will be prospectively carried out by means of a web-based data capture system. The following variables will be recorded upon study enrolment and at follow-up timepoints (Figure 1): sociodemographic data; anthropometric data; lifestyle habits (smoking, alcohol consumption, physical activity); vital signs; clinical history, including information related to T2DM, CVD, CV risk factors and other medical history; information regarding treatment for T2DM, CVD, CV risk factors and exposure to drugs that may cause or exacerbate HF; clinical data (HF symptoms/signs, NYHA functional status, stage and phenotype of HF); functional limitation/quality of life via Kansas City Cardiomyopathy Questionnaire-12; routine hematology, biochemitry and urine testing; 12-lead electrocardiography; cardiac function via echocardiography; serum cardiac biomarkers (NTproBNP, troponin); and CV events of interest after study enrolment (mortality; hospitalizations for HF and for other CV-related reasons; HF-and CV-related HCRU other than hospitalizations).

### 2.5. Study Objectives

The primary outcomes of the study are as follows: the proportion of patients diagnosed with symptomatic HF (Stage C) at enrolment in the overall study population, and the proportions of patients with HF stages A, B and C at enrolment, in the overall study population and based on NYHA functional classification. Select secondary outcomes include the following: the proportion of patients having progressed to higher HF stage at 2 years post-enrolment in the overall study population and in the subpopulations with HF stages A, B and C at enrolment; person-time incidence rate of symptomatic HF (stages C or D) defined as number of patients diagnosed with symptomatic HF per patient-years of observation since enrolment, in the study subpopulation with preclinical HF (stages A and B) at enrolment; proportions of patients with HF with reduced (HFrEF), mildly reduced (HFmrEF) and preserved left ventricular ejection fraction (HFpEF), overall and in the subpopulations with HF stages B and C at enrolment; change in the KCCQ-12 overall summary score and domain scores from enrolment to 6 and 24 months post-enrolment in the study subpopulation diagnosed with symptomatic HF (stage C) at enrolment; and person-time incidence rate of inpatient hospitalizations, emergency room attendances, admissions to an outpatient facility, visits at office-based physicians, home visits by physicians as well as medical procedures/interventions/diagnostic testing utilization for HF- and CV-related causes, in the overall study population and in the subpopulations with HF stages A, B and C at enrolment. A complete list of the secondary and exploratory outcomes of the study is presented in the Supplementary Materials.

### 2.6. Statistical Analysis

This study does not aim to confirm or reject any pre-defined hypotheses; therefore, statistical analysis will mainly be of descriptive and exploratory nature. Inferential statistics will be used for the estimation of the confidence intervals (CIs) of the primary and secondary outcomes (as applicable) as well as in the context of the exploratory outcome analyses. Continuous variables will be summarized using descriptive statistical measures (number of patients with available observations (n_pt_), number of missing observations (n_miss_), mean, standard deviation (SD), median, 25th and 75th percentiles, minimum (min) and maximum (max)), and categorical variables will be displayed using frequency tables (n_pt_, %). The normality of distribution of continuous variables will be examined using the Shapiro–Wilk test. Differences from baseline will be assessed using repeated measures analysis of variance (ANOVA). Concerning binomial proportions, the 95% CIs will be derived from Wald confidence limits for binomial proportions, while for incidence rates, the 95% Poisson CIs will be estimated. Time-to-event analysis will be conducted using the Kaplan–Meier method.

The impact of patients’ characteristics on variables of interest will be evaluated using binary or multinomial logistic regression models for nominal outcome variables. Cox proportional hazard regression models will be applied in order to examine the association of selected factors of interest with the time-to-event outcome variables. Multivariable regression models may be fitted, providing that the number of events/patients in each model is sufficient to allow for meaningful inferences. Missing covariates will be addressed within the appropriate imputation method.

All analyses will be performed in the set of all eligible enrolled patients and in the subpopulations of interest. A comprehensive and detailed description of the statistical methodology for the purposes of the analysis along with imputation methods for missing dates, missing covariates and censoring rules for Kaplan–Meier estimations will be provided in the statistical analysis plan (SAP). All statistical tests will be two-sided, and *p* values will be reported.

## 3. Discussion

The International Diabetes Federation estimated the prevalence of diabetes among Greek adults as 7.4% for 2019; however, accurate epidemiological data are lacking [16]. Patients with type 2 DM have a 2- to 4-fold higher risk than non-diabetics for CV events including HF [17]. The main risk factors for the development of HF in patients with DM include established CV disease, presence of CV risk factors and chronic kidney disease (CKD) [18]. Further to clinical prognosticators, the identification of biomarkers for the prediction and prognosis of HF is an active field of research, with most studies supporting the use of a combination of inflammatory, biochemical and echocardiography biomarkers [19]. There is a need for early identification of patients with T2DM at high risk for progression to HF in order to intervene promptly with medical therapies, aiming not only to provide symptomatic relief but also to delay the development of HF, which will eventually occur in approximately 50% of patients with DM. As a result of the intersection of DM, CVD and HF, the importance of determining diabetes therapies that are not only safe but also effective in reducing CV risk is paramount [20]. Recently, three large randomized trials have shown that sodium-glucose transport protein 2 (SGLT2) inhibitors, used for the treatment of DM, significantly reduce the risk for HF hospitalization [21,22,23]. These studies enrolled patients with DM who have either a history or are at increased risk for developing CVD and showed that the benefits of these medications regarding HF hospitalizations are consistent, independent of a pre-existing diagnosis of HF. As a result, the 2019 Hellenic Diabetes Association (HAD) guidelines, in alignment with the 2019 American Diabetes Association (ADA) guidelines [24], list SGLT2 inhibitors as the recommended treatment option for DM patients with HF or with atherosclerotic CVD at high risk of HF. Moreover, SGLT2 inhibitors have been shown to improve cardiovascular function, as assessed via echocardiography indices and NT-proBNP [25].

## 4. Conclusions 

The HF-LanDMark study is, to our knowledge, the first epidemiological study that will assess the prevalence and 2-year incidence of HF among T2DM patients with no previously diagnosed HF in Greece. We believe that the results will allow for prompt interventions with medical treatments, aiming not only to provide symptomatic relief but also to delay the development of HF in this population.

## Supplementary Materials

The following supporting information can be downloaded at: https://www.mdpi.com/article/10.3390/jcm12196319/s1.
