# Supplementary material for: Rationale and Design of Heart Failure Prevalence and Evolution of Heart Failure in Diabetes Mellitus Type II Patients at High Risk (HF-LanDMark Study)
Source: J Clin Med. 2023 Sep 30;12(19):6319. doi: 10.3390/jcm12196319 (PMC10573953; doi:10.3390/jcm12196319)
Supplement: Supplementary file 1 [file jcm-12-06319-s001.zip › jcm-2590276-supplementary.pdf]

Rationale and design of Heart Failure prevalence and evolution of Heart Failure in Diabetes Mellitus type II patients at high risk (HF-LanDMark study)

Supplementary Materials

Table of contents

1. HF-LanDMark Site Investigators/ Site Coordinators……………………………………………………2
2. Inclusion criteria…………………………………………………………………………………………….3
3. Exclusion criteria……………………………………………………………………………………………5
4. Primary outcome……………………………………………………………………………………………..6
5. Secondary outcomes…………………………………………………………………………………………7
6. Exploratory outcomes……………………………………………………………………………………….9

1. HF-LanDMark Site Investigators/ Site Coordinators

Attikon University Hospital: John Parissis, Christos Georgiou, Vasiliki Bistola, Effie Polyzogopoulou, Antonis Boultadakis, Georgia Psarrou; **Hippokration General Hospital Athens**: Charalambos Vlachopoulos, Ioanna Dima; **"G. Gennimatas" Athens General Hospital**: Apostolos Karavidas, Despoina Balta; **Hippokration General Hospital of Thessaloniki**: Vassilios Vassilikos, Chalil Tsavousoglou, Christodoulos Papadopoulos, Constantinos Bakogiannis, Maria Toumberleka; **Alexandra General Hospital**: John Kanakakis, Argyrios Ntalianis, Maria Tselegkidi, Alexia Mareti; **Department of Cardiology, University of Patras**: Periklis Davlouros, Grigorios Tsigkas, Eleni Koufou; **Cardiology Department, Democritus University of Thrace**: Dimitrios Tziakas, George Chalikias, Vassiliki Tsaliki, Alexandros Markakis; **Evangelismos Hospital**: Ioannis Alexanian, Sotirios Xydonas, Nikolaos Mageiras, Sofia Petropoulou, Georgios Patavoukas; **Heraklion University Hospital**: George Kochiadakis, Eleftherios Kallergis, Ioannis Anastasiou, Emmanouel Kanoupakis; **Larissa University General Hospital**: Filippos Triposkiadis, Michail Papamichalis, Apostolos Dimos, Pinelopi Karagogou; **AHEPA Hospital**: Haralambos Karvounis, George Giannakoulas, Theodoros D. Karamitsos, Georgios Deligiannis, Sofia-Anastasia Mouratoglou, Emmanouela Peteinidou.

2. Inclusion Criteria

- Female or male outpatients aged 40 to 80 years (inclusive) at the time of providing informed consent.
- T2DM diagnosis within 10 years prior to enrollment.
- Patients must have a high or very high CV risk, as outlined in 2019 ESC Guidelines on diabetes, pre-diabetes and CV diseases. *(Cosentino F, Grant PJ, Aboyans V, et al. 2019 ESC Guidelines on diabetes, pre-diabetes, and cardiovascular diseases developed in collaboration with the EASD. Eur Heart J 2020;41:255-323).*

<u>*Note:*</u>

- ***Very high*** *CV risk is defined as having either established CVD*[a]*, or other target organ damage*[b]*, or at least three major risk factors*[c]
- ***High*** *CV risk is defined as age ≥ 50 years and presence of at least one additional major risk factor*

a. Established CVD *defined as:*

- *Ischemic heart disease (IHD) (any of the following): documented myocardial infarction (MI); percutaneous coronary intervention (PCI); coronary artery bypass grafting (CABG); objective findings of coronary stenosis (>50%) in at least 2 coronary arteries*

- *Cerebrovascular disease (CeVD) (any of the following): documented ischemic stroke (known transient ischemic attack, primary intracerebral hemorrhage or subarachnoid hemorrhage do not qualify); carotid stenting or endarterectomy*
- *Peripheral arterial disease (PAD) (any of the following): peripheral arterial stenting or surgical revascularization; lower extremity amputation as a result of peripheral arterial obstructive disease; current symptoms of intermittent claudication and ankle/brachial index <0.90 documented within the past 12 months.*

b. ***Other target organ damage*** *is defined as proteinuria, LVH, or retinopathy*

c. ***Major risk factors*** *include age, hypertension, dyslipidemia, smoking, and obesity (body mass index [BMI]≥30 kg/m^2)*

- *Age ≥50 years*
- *Dyslipidemia is defined as low-density lipoprotein cholesterol (LDL-C)>130 mg/dL with the past 12 months and/or on lipid lowering therapy prescribed by a physician for dyslipidemia*
- *Hypertension, defined as blood pressure>140/90 mmHg at enrollment and/or on antihypertensive therapy prescribed by a physician for blood pressure lowering*
- *Smoking is defined as tobacco use of at least 5 cigarettes/day for at least 12 months prior to enrollment.*

- Patients must have available medical records for data abstraction to meet the objectives of the study.
- Written signed and dated informed consent.

3. Exclusion Criteria

- Diagnosis of type 1 DM.
- Treatment with SGLT2 inhibitor at the time of providing informed consent.
- History of HF (defined as current or previous receipt of treatment prescribed for the management of HF, and/or hospitalization for HF at any time in the past).
- Patients with limitations in their functional capacity that, as per the physician's judgment, are attributed to non-cardiac medical reason or condition (such as lung disease, musculoskeletal disorders, infection, endocrine disorders etc.).
- History of any malignancy within the 5-year period prior to enrollment (with the exception of successfully treated non-melanoma skin cancers).
- Chronic cystitis and/or recurrent genital or urinary tract infections (3 or more in the last year).
- Acute kidney injury or rapidly progressing renal disease.
- Severe renal impairment (estimated glomerular filtration rate [eGFR] <30 mL/min/1.73m^2 using the CKD-EPI equation).
- Hematuria (confirmed by microscopic evaluation) with no explanation as judged by the study physician.

- HbA1c level ≥12%.
- Aspartate aminotransferase (AST) or alanine aminotransferase (ALT) >3 x upper limit of normal (ULN) or total bilirubin >2.5xULN.
- Pregnant or breastfeeding patient.
- Patients with lifetime expectancy less than 2 years due to any non-CV cause or with any non-cardiac condition which, in the judgment of the Investigator, may render the patient unable to complete the study.
- Patients who are currently receiving treatment with any investigational drug/device/intervention or have received any investigational product within 1 month or 5 half-lives of the investigational agent (whichever is longer) prior to enrollment.
- Patients who have already been enrolled in this study or in a study of the same design which the physician has good reasons to believe that it is this study (to exclude enrollment of the same patient by two different sites).

4. Primary Outcome

- Proportion of patients diagnosed with symptomatic HF (Stage C) at enrollment in the overall study population.

- Proportions of patients with HF Stage A, Stage B, and Stage C at enrollment, in the overall study population and by NYHA functional classification.

5. Secondary Outcomes

- Proportion of patients having progressed to higher HF stage at 2 years post-enrollment in the overall study population and in the subpopulations with HF Stage A, Stage B, and Stage C at enrollment.
- Proportion of patients having progressed to higher NYHA functional class at 2 years post-enrollment in the overall study population.
- Proportion of patients having progressed to higher NYHA functional class at any time post-enrollment during the study observation period in the overall study population.
- Person-time incidence rate of symptomatic HF (Stage C or D) defined as number of patients diagnosed with symptomatic HF per patient-years of observation since enrollment, in the study subpopulation with preclinical HF (Stages A and B) at enrollment.
- 2-year cumulative incidence rate (estimated by the use of time-to-event analysis methods) of progression to symptomatic HF, in the study subpopulation with preclinical HF at enrollment.
- Person-time incidence rate of symptomatic HF defined as number of patients with symptomatic HF per patient-years of observation since enrollment, in the study subpopulation with HF Stage B at enrollment.
- 2-year cumulative incidence rate (estimated by the use of time-to-event analysis methods) of progression to symptomatic HF among patients with HF Stage B at enrollment.

- Summary statistics of clinical and echocardiographic characteristics as well as of cardiac circulating biomarkers, overall and by HF stage at enrollment.
- Proportion of patients with abnormal cardiac circulating biomarkers (NT-proBNP and hs-cTn), overall and by HF stage at enrollment.
- Proportions of patients with HFpEF, HFmrEF, and HFrEF, overall and in the subpopulations with HF Stage B and HF Stage C at enrollment.
- Proportion of patients with abnormal echocardiographic GLS (i.e, absolute value <16%), overall and in the subpopulations with HF Stage B and HF Stage C at enrollment.
- KCCQ-12 overall summary score and domain (physical limitation, symptom frequency, quality of life and social limitation) scores at enrollment and at 6 and 24 months post-enrollment, in the study subpopulation of patients diagnosed with symptomatic HF (Stage C) at enrollment.
- Change in the KCCQ-12 overall summary score and domain scores from enrollment to 6 and 24 months post-enrollment in the study subpopulation diagnosed with symptomatic HF (Stage C) at enrollment.
- Person-time incidence rate of inpatient hospitalizations, emergency room attendances, admissions to an outpatient facility, visits at office-based physicians, home visits by physicians as well as of medical procedures/interventions/diagnostic testing utilization for HF- and CV-related causes, in the overall study population and in the subpopulations with HF Stage A, Stage B, and Stage C at enrollment.
- Summary statistics of inpatient length of stay (LOS) (overall LOS, intensive care unit/high dependency unit [ICU/HDU] LOS, and non-ICU/HDU LOS) for HF- and CV-

related causes over the 2-year study observation period, in the overall study population and in the subpopulations with HF Stage A, Stage B, and Stage C at enrollment.

6. Exploratory Outcomes

- Association of baseline clinical, echocardiographic and biochemical parameters with the HF stage at enrollment.
- Association of baseline clinical, echocardiographic and biochemical parameters with the presence of symptomatic HF at enrollment.
- Association of baseline clinical, echocardiographic and biochemical parameters with progression to symptomatic HF over the 2-year observation period in the study subpopulation with preclinical HF at enrollment.
- Association of changes in NT-proBNP levels between enrollment and 6 months with subsequent progression of HF (i.e., transition to a higher stage compared to 6 months) in the overall study population.
- Association of changes in NT-proBNP levels between enrollment and 6 months with subsequent occurrence of CV events of interest and hospitalization for heart failure over the study observation period.
- Proportion of patients with an episode of acute kidney injury, defined as at least doubling of serum creatinine levels from previous visit.

- Change in eGFR from baseline over the 2-year observation period in the overall study population and in the subpopulations with HF Stage A, Stage B, and Stage C at enrollment.
